# Cortical Adaptation to Muscle Fatigue Does Not Alter Early Proprioceptive Processing in Primary Sensorimotor Cortex

**DOI:** 10.1111/ejn.70742

**Published:** 2026-11-02

**Authors:** Junru Chen, Toni Mujunen, Feiyue Li, Riku Nikander, Harri Piitulainen

**Affiliations:** ^1^ Faculty of Sport and Health Sciences University of Jyväskylä Jyväskylä Finland; ^2^ Center for Interdisciplinary Brain Research University of Jyväskylä Jyväskylä Finland; ^3^ Center for Neuroplasticity and Pain, Department of Health Science and Technology Aalborg University Aalborg Denmark; ^4^ Wellbeing Services County of Central Finland Jyväskylä Finland

**Keywords:** cortical inhibition, corticokinematic coherence, fatigability, magnetoencephalography, proprioception, somatosensory

## Abstract

Muscle fatigue potentially interferes with proprioceptive afference from peripheral “movement sensors”—the proprioceptors, which may hinder crucial sensorimotor integration and thus movement performance. However, little is known about how muscle fatigue affects cortical processing of proprioceptive afference. Twenty‐four healthy volunteers (30.7 ± 6.5 years) participated in the experiment, which included magnetoencephalography (MEG) recordings during 2‐Hz passive ankle‐joint rotations, and fatigue tasks comprised of isometric ankle plantarflexion. Corticokinematic coherence (CKC) between linear foot acceleration and MEG signals was examined before (PRE) and ~3 min after (POST) the fatigue tasks to compare the cortical proprioceptive processing. CKC peaked in the gradiometer pairs above the foot region of the primary sensorimotor (SM1) cortex in each participant. CKC strength did not show significant difference between PRE and POST at 2 Hz (0.30 ± 0.12 vs. 0.30 ± 0.14, *p* = 0.981) or its first harmonic at 4 Hz (0.38 ± 0.14 vs. 0.37 ± 0.13, *p* = 0.724). However, 4‐Hz MEG power was ~30% lower in POST than in PRE. Surprisingly, fatigue‐induced increase of alpha and beta power was observed bilaterally in SM1 hand regions during the ankle movements. Our results indicate that the early processing of proprioceptive afference from the ankle joint was negligibly affected by muscle fatigue or the effect recovered rapidly. The fatigue elevated alpha and beta power while diminishing neuronal activity at the 4‐Hz harmonic frequency, which may reflect a compensatory mechanism for asynchronous proprioceptive afference by enhancing cortical inhibitory control.

## Graphical abstract

Cortical proprioceptive responses to passive ankle‐joint movement are negligibly affected by muscle fatigue. Intriguingly, fatigue‐induced increases in alpha and beta power extend beyond the SM1 foot region to bilateral hand regions, which may facilitate more focused lower‐limb proprioceptive processing, thus compensating for asynchronous proprioceptive afference caused by fatigue.

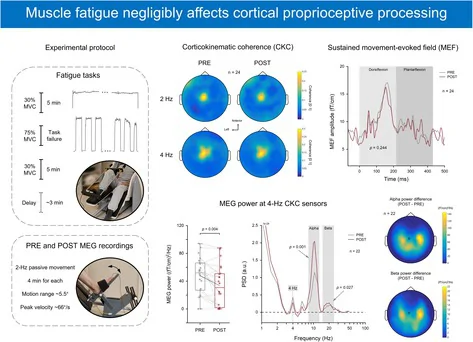

**Abbreviations**
CKC: corticokinematic coherence
CoV: coefficient of variation
EMG: electromyography
GM: gastrocnemius medialis
MDF: median frequency
MEF: movement‐evoked field
MEG: magnetoencephalography
MVC: maximal voluntary contraction
PSD: power spectral density
RMS: root mean square
SM1: primary sensorimotor cortex
SOL: soleus
TA: tibialis anterior

## 1 Introduction

Muscle fatigue in the lower extremities primarily affects balance and locomotor performance (Helbostad et al. 2010; T. Li et al. 2024; Parijat and Lockhart 2008), both of which rely on proprioception. Proprioception encompasses various senses of body position and movement through afferent information arising from those mechanosensitive receptors called proprioceptors (Proske and Gandevia 2012; Tuthill and Azim 2018). It has been widely acknowledged that muscle fatigue can alter proprioceptive perception of the ankle joint, as evidenced by changes in the detection of motion (Lin et al. 2008), joint‐position sense (Allen et al. 2010), and force sense (Brezovar et al. 2025; Proske and Allen 2019). The mismatch between perceived exertion and actual force output during fatigue suggests that muscle fatigue may alter the integration of motor command and peripheral somatosensory feedback (Smith et al. 2007; Søgaard et al. 2006; Taylor et al. 2006). However, there is no evidence on how cortical processing of proprioception itself is altered by muscle fatigue.

Proprioceptors are highly sensitive to forces applied to the muscles, tendons, ligaments, and connective tissues surrounding the joints in a manner like cutaneous mechanoreceptors to touch. For example, the most sensitive ending of muscle spindles can detect below 10‐μm changes in the muscle length (Brown et al. 1967; Roll et al. 1989). Golgi tendon organs on the other hand are activated with the rise of muscle tension (Fallon and Macefield 2007; Jami 1992). Various joint receptors adaptively respond to stretch acting on the joint capsule and firing tonically near the extremes of joint range of motion (V. G. Macefield 2021; Rossi and Grigg 1982). Proprioceptive afference, originating from the periphery, traverses the spinal cord towards the thalamic circuits and subsequently reaches the cerebral cortex via the dorsal column‐medial lemniscus pathway (Tuthill and Azim 2018). The proprioceptive input projects extensively to the somatosensory and motor cortices, with the most dense input to the primary sensorimotor (SM1) cortex contralateral to the peripheral proprioceptors (Ciccarelli et al. 2005).

Acute fatigue in muscles can alter firing properties of the proprioceptors. Evidence from anesthetized cats indicates that muscle fatigue can enhance group Ia and II afferent reactivity to passive stretch and vibration, such as increased firing rate and shortened stretch response latency (Nelson and Hutton 1985), while reducing sensitivity to stretch amplitude (Pedersen et al. 1998). In humans, however, prolonged isometric contraction leads to a progressive decline in spindle firing, which was observed in 72% of recorded afferents (G. Macefield et al. 1991). Importantly, direct manipulation of Ia afferents via superimposed tendon vibration does not alleviate muscle fatigue during high‐intensity intermittent isometric contractions (Timbert et al. 2025). Moreover, postexercise modulation of cortical excitability is partly mediated by residual afferent firing, such as group Ib and II afferents that are sensitive to intramuscular tension and static fiber length, in relaxed muscles (Gruet et al. 2013).

Furthermore, the fatigue‐related changes in somatosensory afference also occur in the brain. It has been reported that beta and gamma cortex‐muscle coupling in ascending direction increased in the presence of isometric grip fatigue (Bedard et al. 2025; Liang et al. 2021). In numerous postexercise electroencephalography (EEG) studies, increased alpha and beta activity was observed in the bilateral parietal cortex (Hosang et al. 2024), as well as magnetoencephalography (MEG) evidence from upper‐limb fatigue studies (Fry et al. 2017; Tecchio et al. 2006), but this exercise‐induced modulation was suspected to be overall brain oscillations that vanished immediately after the cessation of the exercise (Ciria et al. 2018). As is well known, the sensorimotor rhythms that are recorded over the Rolandic regions, typically 8–40 Hz in frequency, can be modulated by limb movements and tactile stimulation (Hari and Salmelin 1997). For example, active movements with varying duration modulate sensorimotor rhythms to different degrees (Cassim et al. 2000; Pakenham et al. 2020), and even brief passive movement is robust modulator of ~20‐Hz rhythm (Parkkonen et al. 2015). Cortical responses to passive movement stimuli mainly reflect proprioceptive afference because they remain observable even in the absence of cutaneous feedback (Abbruzzese et al. 1985; Mima et al. 1996).

The cortical processing of proprioceptive afference is noninvasively assessed through corticokinematic coherence (CKC), which refers to the synchrony between cortical neuronal activity—measured with MEG or EEG—and limb kinematics (Bourguignon et al. 2011, 2015; Piitulainen et al. 2013b, 2020). The strength of CKC typically peaks at the movement frequency and its harmonics. In fact, periodic limb kinematics is not a sinusoidal signal due to rapid deceleration and acceleration around the onsets of movement (Antonsson and Mann 1985). According to the principles of the Fourier transform, any periodic signal that is not a perfect sine wave will transfer energy to its harmonics. The strongest CKC occurs simultaneously with the most regular movement stimuli (Mujunen et al. 2021), and it can be amplified with more comprehensive stimulation of homologous extremities, for example, several fingers versus single one (Hakonen et al. 2022). Furthermore, CKC can be reliably estimated based on any peripheral signal picking the rhythmicity of movement (Piitulainen et al. 2013a). In the present study, proprioceptive stimulation was elicited on the ankle joint by means of rhythmic passive movement at 2 Hz.

To examine the effect of muscle fatigue on early cortical proprioceptive processing, we quantified CKC strength and respective sustained movement‐evoked field (MEF) amplitude during passive ankle movement before and after isometric fatigue tasks. There is growing evidence from fMRI (Nurmi et al. 2021), MEG (Piitulainen, Seipäjärvi, et al. 2018), and EEG studies (Legrand et al. 2026) that intensified cortical proprioceptive processing is associated with worse motor performance, indicating that more cortical neurons are involved in the proprioceptive processing in those individuals with worse sensorimotor function. This association seems to be a cortical compensation mechanism for less optimized sensorimotor integration. Thus, we hypothesized that effects of muscle fatigue on somatosensory afference and sensorimotor integration would be compensated by allocating more cortical resources, that is, neurons in the associated neuronal networks, to adapt to the fatigued state. Therefore, we expected to observe stronger CKC and MEF amplitude following the fatigue protocol. In addition to CKC and MEF, we examined whether the muscle fatigue increases the sensorimotor alpha (~10 Hz) and beta (~20 Hz) power, as has been previously shown to occur after fatiguing exercise (Hosang et al. 2024). The increase in SM1 alpha and beta power is suggested to reflect stronger neuronal synchronization through inhibitory control of local neural populations (Jensen et al. 2005; Jensen and Mazaheri 2010). Thus, we hypothesized both the alpha and beta power to be increased following the fatigue protocol.

## 2 Materials and Methods

### 2.1 Participants

Twenty‐four healthy adults (age 30.7 ± 6.5 years., BMI 23.3 ± 3.6, mean ± SD; 13 females) with no known history of neurological or musculoskeletal disorders were recruited for the study. Eighteen participants were right‐footed and six were mixed‐footed based on the Waterloo Footedness Questionnaire (Grouios et al. 2009). Prior to their enrollment, participants were requested to sign a written informed consent after a necessary understanding of the procedure. The experimental protocol was approved by the Ethics Committee of the University of Jyväskylä (Approval Number 633/13.00.04.00/2024), and the experiments were carried out in accordance with the Declaration of Helsinki.

The sample size was calculated for the within‐subject comparison of CKC before and after fatigue. In the absence of directly comparable prior effect size estimates, a moderate effect of 0.6 was assumed, informed by an earlier study demonstrating large between‐group effects (*d* = 1.16) on CKC values (Piitulainen, Seipäjärvi, et al. 2018). Based on these assumptions, a sample size of 24 provides 80% statistical power for this comparison at *α* = 0.05 significance level.

This study conducted a within‐subject design because the cortical proprioceptive processing, for example, CKC (J. Chen et al. 2025; Piitulainen, Illman, et al. 2018; Piitulainen et al. 2020) and movement‐evoked responses (Illman et al. 2022; Mujunen et al. 2022), have been validated as reproducible in longitudinal measurements. The within‐subject comparison allowed each participant as their own control before and after fatigue, which can minimize the confounding effects of interindividual anatomical and neurophysiological variances in MEG recordings.

### 2.2 Experimental Procedure

The measurements were conducted at the MEG lab in the Centre for Interdisciplinary Brain Research, University of Jyväskylä, Jyväskylä, Finland. Participants were first familiarized with the MEG environment and experimental devices. Then, a warm‐up consisting of four 30‐s in‐place hopping was performed, with 30‐s rest intervals in between. A custom‐made MEG‐compatible device with a load transducer was used to perform fatigue tasks and measure ankle plantar force. The right foot was adjusted to its preferred position where the ankle joint was maintained ~100° throughout the subsequent isometric contractions. The isometric maximal voluntary contraction (MVC) force was defined as the highest value among three utmost attempts, each lasting for ~3 s. Figure 1 illustrates the experimental setup and procedures.

**FIGURE 1:**
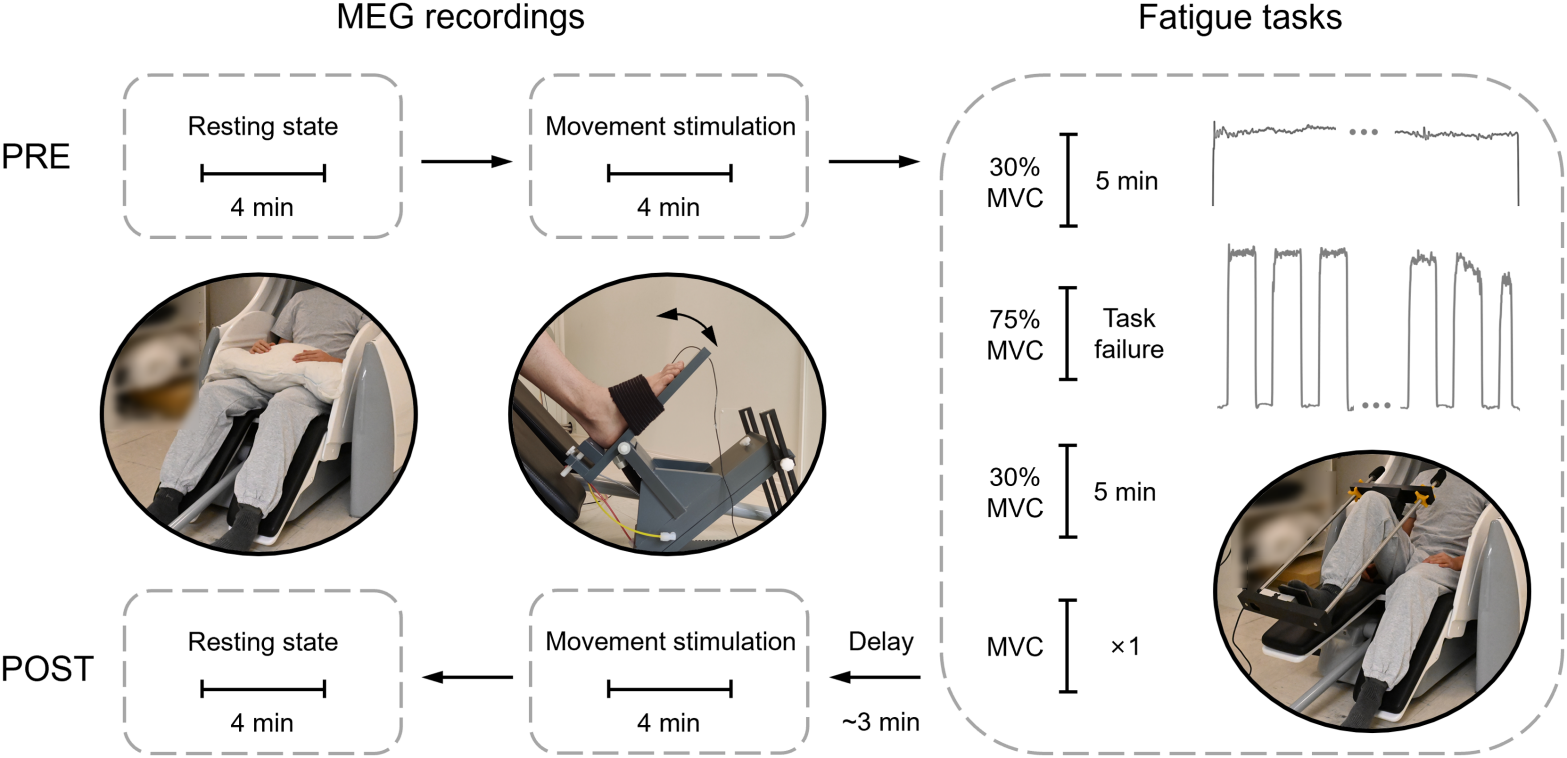
Experimental protocol and the device setup. For the resting state, participants were sitting comfortably in the MEG chair with their arms placed on the pillow in their laps and fixated on a projected focal image on the screen. For the movement stimulation, participant's right foot was fastened to the pneumatic actuator with elastic straps, otherwise identical to the resting. For the fatigue protocol, participants were instructed to adjust to their preferred position and maintain it throughout the tasks whenever possible. The force signals collected from a representative participant during steady and intermittent isometric contractions were respectively shown in the right panel. The delay means approximately 3‐min recovery period after fatigue tasks.

#### 2.2.1 Fatigue Protocol

Muscle fatigue was induced through sustained submaximal isometric plantarflexion of the right ankle. Figure 1 right panel shows the fatigue protocol. The fatigue tasks consisted of two sustained 30% of MVC isometric contractions for 5 min each, and in between, a series of intermittent 75% of MVC isometric contractions. Participants performed 75% of MVC contractions 13 ± 4 (range 11–20) times, each lasting 20 s and separated by 10‐s rests, until they failed to reach the target force. The target force levels were visualized using Signal software version 8 (Cambridge Electronic Design Ltd., Cambridge, UK) projected on a translucent screen placed 0.9 m in front of them. Participants were instructed to generate isometric force as steady as possible on a dotted horizontal line within ±5% of MVC boundaries presented with solid lines. Upon the completion of the fatigue protocol, the force device was immediately replaced with the proprioceptive stimulator as quickly as possible, and the movement stimulation began ~3 min (on average 228 ± 34 s) after MVC test.

#### 2.2.2 Proprioceptive Stimulation

Proprioceptive stimuli (i.e., ankle‐joint rotations) were generated using a movement actuator with pneumatic artificial muscles that are controlled by computer‐generated trigger pulses (Piitulainen, Seipäjärvi, et al. 2018). The stimulation program was performed in Presentation software version 21.1 (Neurobehavioral Systems Inc., Albany, CA, USA). Participant's right foot was placed on the actuator, with the ankle secured in position using elastic straps. The movement actuator induced continuous passive dorsiflexion of the ankle joint (rotation range ~5.5°, peak angular velocity 66°/s) at 2‐Hz movement frequency. Proprioceptive stimuli were delivered to the ankle joint for 4 min, respectively, before (PRE) and after the fatigue tasks (POST). Participants were asked to fixate on a focal image through a rectangular hole in the cardboard in front of them. To minimize the auditory involvement of airflow noise from the actuator, we provided earplugs to participants and played 70‐dB Brownian noise through flat panel speakers. In addition, resting‐state MEG signals were recorded at the beginning and end of the MEG session for 4 min each.

### 2.3 Data Acquisition

The MEG recordings were carried out in a magnetically shielded room with a 306‐channel Vectorview neuromagnetometer (Elekta Neuromag TRIUXTM, Elekta Oy, Helsinki, Finland) that contains 102 magnetometers and 204 planar gradiometers. MEG signals were band‐pass filtered online at 0.1–330 Hz and sampled at 1000 Hz. Foot kinematics was recorded using three‐axis linear accelerometers (ADXL335 iMEMS Accelerometer, Analog Devices Inc., Norwood, MA, USA) attached to the first proximal phalanx of the foot. The acceleration signals were lowpass‐filtered at 330 Hz and sampled at 1000 Hz, time‐locked to MEG signals. Synchronously, the digital trigger signals were also recorded to correct time axis for a fixed 65‐ms pneumatic delay of the actuator from the trigger onset to the actual movement.

The plantar force during fatigue tasks was measured with a rigid load cell (model YT‐108, capacity 1962 N; Yongtai Measuring Instrument Co. Ltd., Hangzhou, China) mounted on the custom‐made device. Muscle activity was quantified using surface electromyography (EMG) recorded with Ag/AgCl electrodes (Ambu Neuroline 720, Ambu A/S, Ballerup, Denmark) taped on the tibialis anterior (TA), gastrocnemius medialis (GM) and soleus (SOL) muscles. The recording passband was 0.1–330 Hz for EMG signals and DC–330 Hz for force signal. EMG and force signals were then sampled at 1000 Hz, both time‐locked with MEG signals.

### 2.4 Data Processing

#### 2.4.1 Force and EMG Signal Processing

Force signals were first band‐pass filtered at 0.1–20 Hz with fourth‐order Butterworth filter. Only the stable segments of force signals within the ±5% target boundaries were included in further analyses. Force steadiness was quantified by the coefficient of variation (CoV) of force, calculated as the ratio of the standard deviation to the mean.

The raw EMG signals were time aligned with the selected force segments. EMG signals were band‐pass filtered at 5–295 Hz with fourth‐order Butterworth filter. The root mean square (RMS) was then calculated using 250‐ms sliding windows with 50% overlap. Median frequency (MDF) was estimated using Welch's method with 1‐s sliding windows and 50% overlap.

#### 2.4.2 MEG Signal Preprocessing

MEG raw data were first preprocessed using the temporal signal space separation (tSSS; Taulu and Simola 2006) to suppress external interferences and correct for head movements in MaxFilter software version 3.0.17. The data were further preprocessed with MNE‐Python software (Gramfort et al. 2013) to suppress physiological noise. After band‐pass filtering the data to 1–40 Hz with a zero‐phase finite impulse response filter (firwin in SciPy; FIR filter design using Hamming window), a fast independent component analysis (ICA; Hyvarinen 1999) was implemented to decompose the data into 30 independent components. Then, two to four ICA components corresponding to eye movements and cardiac activities were identified and ultimately discarded from the raw data.

#### 2.4.3 Coherence Analysis

Coherence analysis was carried out on the sensor level. Continuous MEG data were split into 2‐s epochs with 80% overlap, leading to a frequency resolution of 0.5 Hz (Bortel and Sovka 2007). Epochs with the signals exceeding 3 pT for magnetometers or 0.7 pT/cm for gradiometers were excluded to avoid contamination by any artifact not removed by the preprocessing. Next, coherence analysis (Halliday et al. 1995) was performed—yielding power, cross‐, and coherence spectra, as well as correlograms—between all MEG sensors and the Euclidian norm of the three orthogonal accelerometer signals. The number of artifact‐free epochs (560 ± 45, mean ± SD; range 483–595) was fixed equally for each participant between the two CKC recordings. The signals from gradiometer pairs were combined in the direction of maximum coherence (Bourguignon et al. 2015), and then, the gradiometer pair with the highest coherence value was selected from 10 gradiometer pairs over the left Rolandic foot region.

#### 2.4.4 MEG Steady‐State Field Analysis

MEG signals were first split into 1.5‐s epochs, and then averaged with respect to the trigger onsets of evoked movements for each participant and condition. The resulting steady‐state field was referred to as the sustained MEF. The peak‐to‐peak amplitude of MEF was calculated and finally extracted from the gradiometer pair that showed peak CKC at 2 Hz. The actual movement onset was defined as the time when the Euclidian acceleration reached 15% of its peak magnitude. Thus, about 10 ms before the first and second acceleration peaks, the dorsiflexion and plantarflexion phases respectively commenced.

#### 2.4.5 Power Spectral Analysis of MEG Signals

Power spectral density (PSD) for the passive movement was computed concurrently with the coherence analysis and then combined the vector sum of the gradiometer pair at each sensor location. The power spectrum comprises both narrowband periodic oscillations (such as alpha and beta peaks) and broadband aperiodic activity (Gao et al. 2017). Because muscle fatigue has been shown to alter the overall metabolic state and cortical excitation/inhibition balance (Coxon et al. 2018; Maddock et al. 2016), we separated the periodic oscillations from any global cortical changes, that is, aperiodic activity. The power spectra were decomposed into the periodic and aperiodic component using FOOOF algorithm (Donoghue et al. 2020) with aperiodic mode “knee,” across the frequency range 1–49.5 Hz. Only the fitting results with *R* ^2^ > 0.90 were accepted for further analyses.

After subtraction of the aperiodic component, the remaining PSD was used to calculate the power values at frequencies of interest: alpha band (8–13 Hz), beta band (15–25 Hz), movement frequency (2 Hz), and its first harmonic (4 Hz). In case the peaks of power spectra were lower than the fit, that is, the periodic component at a given frequency is negative, its power was set to zero. The power in alpha band was defined individually for each recording as the mean within ±1 Hz around the frequency with peak power. Similarly, the beta‐band power was the mean within ±2 Hz around the frequency with peak power.

### 2.5 Statistical Analysis

#### 2.5.1 Statistical Significance of Coherence

The statistical significance of the individual coherence levels was assessed under the assumption that the Fourier coefficients were linearly independent from one epoch to the next at each frequency of interest, while accounting for the use of overlapping epochs (Bourguignon et al. 2011; Halliday et al. 1995). To correct for multiple comparisons, the significance threshold was defined as *α* = 0.05/(*N*_*f*_ × *N*_*s*_), of which *N*_*f*_ = 2, being the number of tested frequency bins, and *N*_*s*_ = 10, the number of preselected gradiometer pairs.

#### 2.5.2 Statistical Differences Between PRE and POST

The normality of the differences between paired data was initially assessed using the Shapiro–Wilk test. When the assumption of normality was accepted, paired *t*‐test was used to evaluate statistical differences between paired data before and after fatigue, with effect sizes calculated using Cohen's *d*_*z*_. The magnitude of Cohen's *d*_*z*_ was interpreted as follows: small (0.2–0.5), medium (0.5–0.8), and large (≥ 0.8) (Cohen 1988). For nonnormally distributed data, the nonparametric Wilcoxon signed‐rank test was employed. Statistical significance was considered for all results when two‐tailed *p* < 0.05.

#### 2.5.3 Correlations Between CKC, MEF, and MEG Power

To further estimate the effect of MEG signal‐to‐noise ratio on CKC, correlation analysis was performed for CKC strength with the corresponding MEF amplitude and MEG power. Pearson correlation coefficient (*r*) was used for normally distributed data, whereas for nonnormal distribution, Spearman correlation coefficient (*r*_*s*_) was employed.

## 3 Results

All participants included in the final analysis (*n* = 24) showed significant CKC strength at the movement frequency (2 Hz) and its first harmonic (4 Hz). The 1/*f*‐like component fit of MEG power spectrum was achieved in 22/24 participants during movement stimulation, with the remaining two excluded for poor model fit (*R* ^2^ < 0.90). Thus, 22 participants (age 30.5 ± 6.7 years, 13 females) were included in the power spectral analysis.

### 3.1 The Degree of Muscle Fatigue

Nonfatigued and fatigued conditions were compared using the first 30 s of the first steady isometric contraction and the last 30 s of the second contraction. Figure 2 illustrates the effect of muscle fatigue on force steadiness and muscle activity. As shown in Table 1, the CoV of force significantly increased at the end of fatigue protocol, indicating reduced force steadiness in fatigued condition. The RMS of EMG amplitude increased significantly in TA, GM, and SOL muscles when they were fatigued. For the MDF of EMG signals, significant reductions were observed in TA and SOL following fatigue, but no significant difference was shown in GM. The MVC force was significantly lower immediately after the fatigue protocol (812 ± 134 N) than before fatigue (1044 ± 140 N, *p* < 0.001).

**FIGURE 2:**
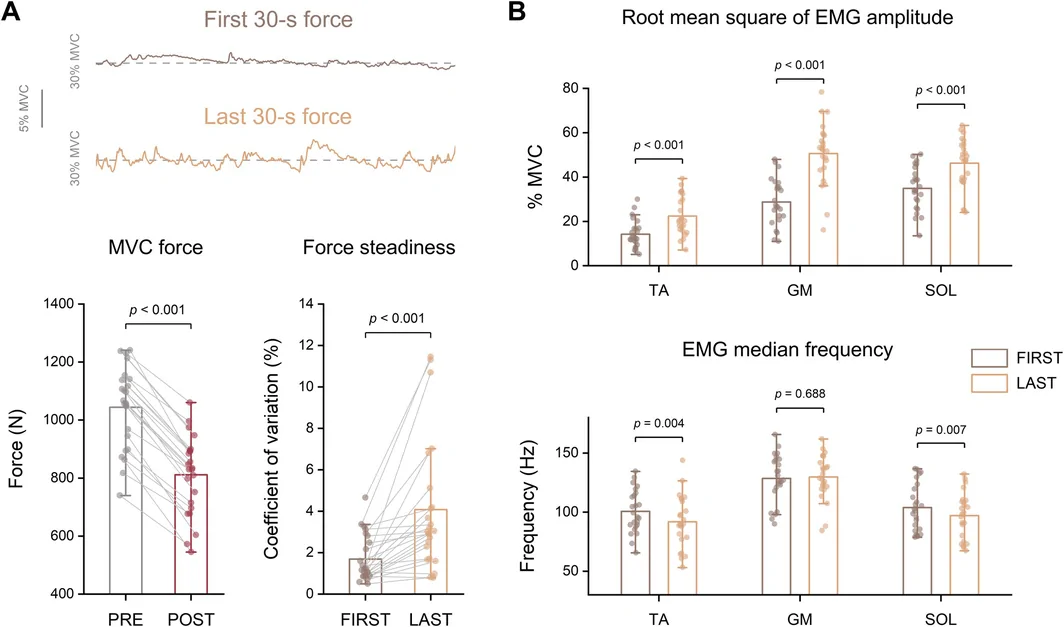
Force and EMG activity indicative of muscle fatigue. (A) Force fluctuations at the first and last 30 s of the fatigue protocol from a representative participant (upper panel). The force output of maximum voluntary contraction (MVC) before and after fatigue and the coefficient of variation of steady isometric force (lower panel). (B) The root mean square of EMG amplitude in the tibialis anterior (TA), gastrocnemius medialis (GM), and soleus (SOL) muscles and their EMG median frequency at the first and last 30 s of the fatigue protocol.

**TABLE 1:** Force steadiness and EMG activity.

		First	Last	*p*
Force CoV, %		1.69 ± 1.12	4.07 ± 3.17	**< 0.001**
RMS, %MVC	TA	14.2 ± 6.3	22.4 ± 8.9	**< 0.001**
	GM	28.8 ± 10.7	50.6 ± 14.1	**< 0.001**
	SOL	34.9 ± 9.8	46.2 ± 11.2	**< 0.001**
MDF, Hz	TA	100.6 ± 18.2	91.8 ± 22.3	**0.004**
	GM	128.5 ± 20.6	129.7 ± 19.0	0.688
	SOL	103.8 ± 20.1	97.1 ± 19.5	**0.007**

*Note:* Values are mean ± SD. Bold indicates a significant difference when *p* < 0.05.

Abbreviations: CoV, coefficient of variation; GM, gastrocnemius medialis; MDF, median frequency; MVC, maximal voluntary contraction; RMS, root mean square; SOL, soleus; TA, tibialis anterior.

### 3.2 Effect of Muscle Fatigue on CKC

Figure 3 presents the CKC strength from the gradiometer pairs showing the peak value at 2 and 4 Hz. The CKC strength did not significantly differ between PRE and POST at either 2 or 4 Hz (Table 2). The grand average topographies showed, as expected, that CKC peaked consistently on the gradiometer pairs over the Rolandic foot area. In 10/24 participants, 2‐ and 4‐Hz CKC peaked in a different sensor, but the sensors were always the adjacent ones. The peak CKC sensor was largely consistent from PRE to POST recordings, with only 2/24 participants showing a shift to the adjacent sensor because of unintended minor changes in head position. One participant showed nonsignificant 2‐Hz CKC of 0.04 in POST, while the corresponding value in PRE was 0.15.

**FIGURE 3:**
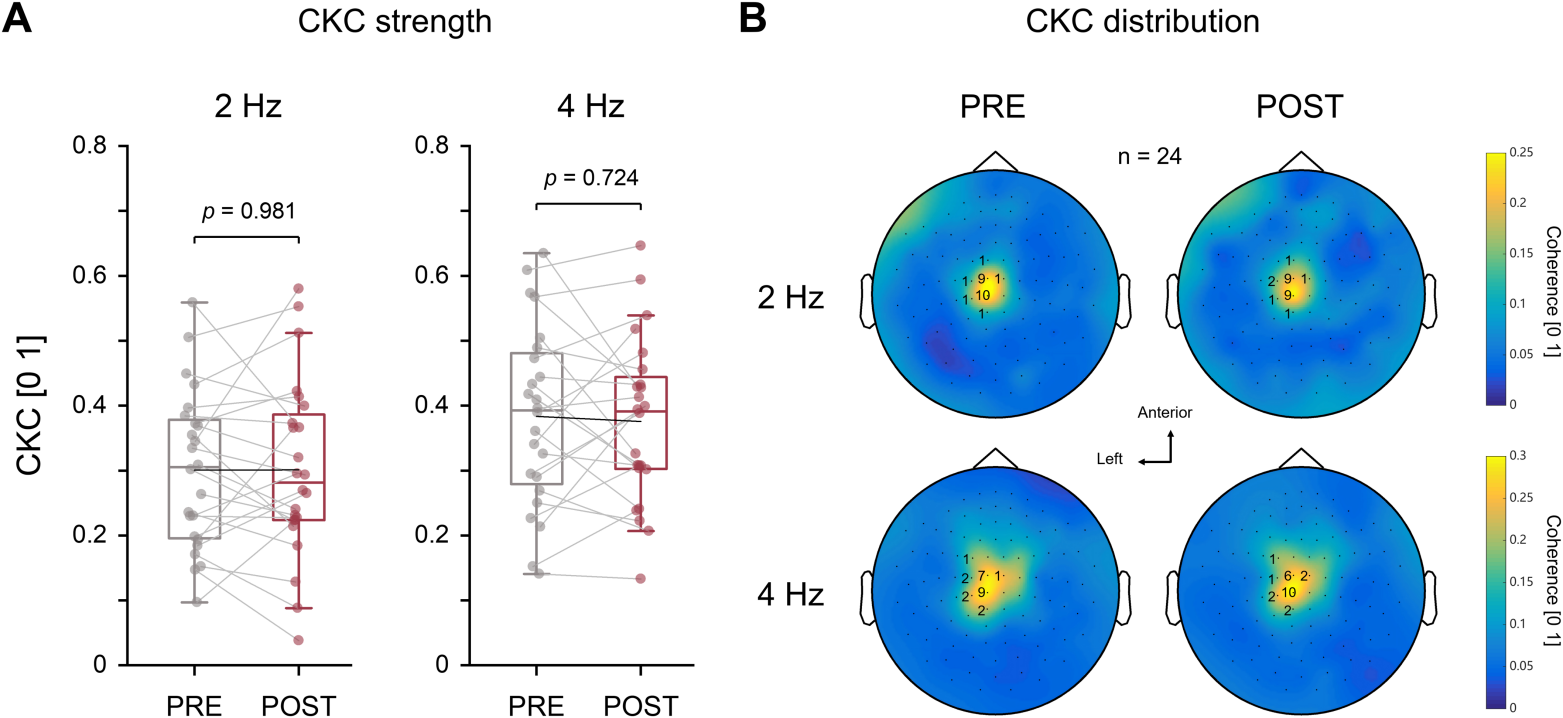
CKC strength and its topographic distributions (*n* = 24) before (PRE) and after fatigue (POST). (A) Peak CKC at 2 and 4 Hz. Solid black lines connect the group mean values. The horizontal edges of the box indicate the interquartile range of the distribution, and the horizontal line in the box indicates the median. (B) The grand average topographies of 2‐ (upper panel) and 4‐Hz CKC (lower panel). The number of peak CKC gradiometer pairs is marked in black.

**TABLE 2:** Cortical responses to movement stimulation.

		PRE	POST	*p*	Cohen's *d*_*z*_
CKC strength	2 Hz	0.30 ± 0.12	0.30 ± 0.14	0.981	0.01
	4 Hz	0.38 ± 0.14	0.37 ± 0.13	0.724	0.06
MEF amplitude, fT/cm		22.8 ± 6.3	21.2 ± 7.1	0.244	**0.24**
MEG power, (fT/cm)^2^/Hz	2 Hz	97.9 ± 57.1	91.9 ± 67.4	0.570	0.10
	4 Hz	47.8 ± 26.7	33.6 ± 29.1	**0.004**	**0.51**

*Note:* Values are mean ± SD. Bold indicates a significant difference when *p* < 0.05, or a substantive effect when Cohen's *d_z_* > 0.2.

Abbreviations: CKC, corticokinematic coherence; MEF, movement‐evoked field; MEG, magnetoencephalography.

### 3.3 Effect of Muscle Fatigue on Sustained MEF

Figure 4A shows sustained MEF that extracted from the gradiometer pairs showing strongest 2‐Hz CKC. The sustained MEF amplitude did not significantly differ between PRE and POST (Table 2). Figure 4B illustrates the scatter plots and correlation between CKC strength and MEF amplitude. In PRE recording, stronger sustained MEF was associated with stronger CKC, accounting for ~30% of the shared variance. However, in POST recording, the above correlative relation was no longer statistically significant.

**FIGURE 4:**
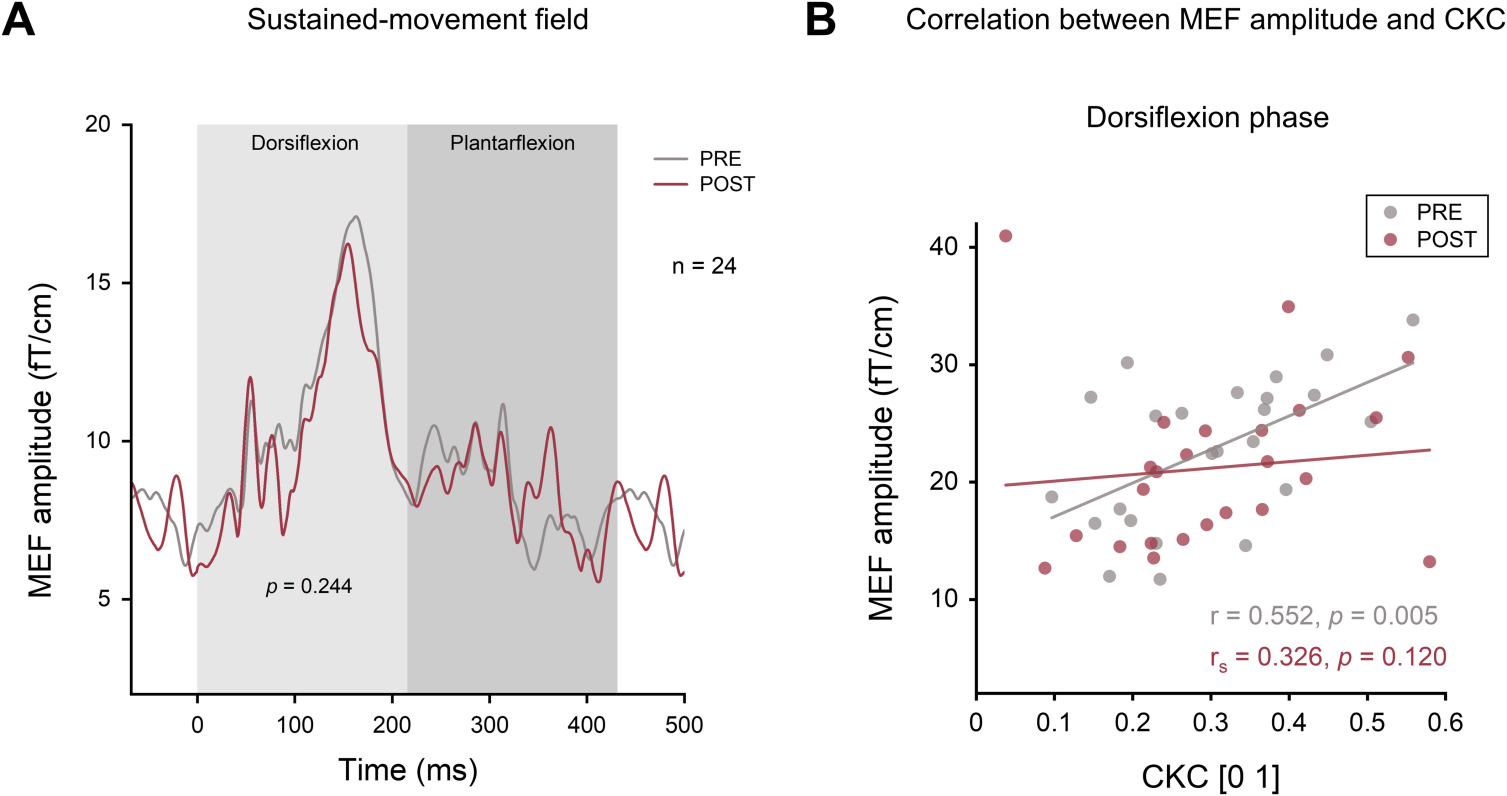
Sustained movement‐evoked field (MEF) before (PRE) and after fatigue (POST). (A) Grand averages of MEF (*n* = 24) from the peak 2‐Hz CKC sensors. The dorsiflexion and plantarflexion movement phases are highlighted with light and dark gray, respectively. The onset of movement stimulus is the beginning of dorsiflexion phase. (B) Correlation between 2‐Hz CKC strength and the corresponding MEF amplitude.

### 3.4 MEG Power Modulation to Muscle Fatigue in Peak CKC Sensors

Figure 5 presents MEG power spectra extracted from the peak CKC sensors at 2 and 4 Hz. MEG power at 2 Hz showed no significant difference between PRE and POST, while MEG power at 4 Hz was significantly weaker in POST compared with PRE (Table 2). CKC strength showed a significant correlation with both 2‐ and 4‐Hz power, both in PRE and POST recordings.

**FIGURE 5:**
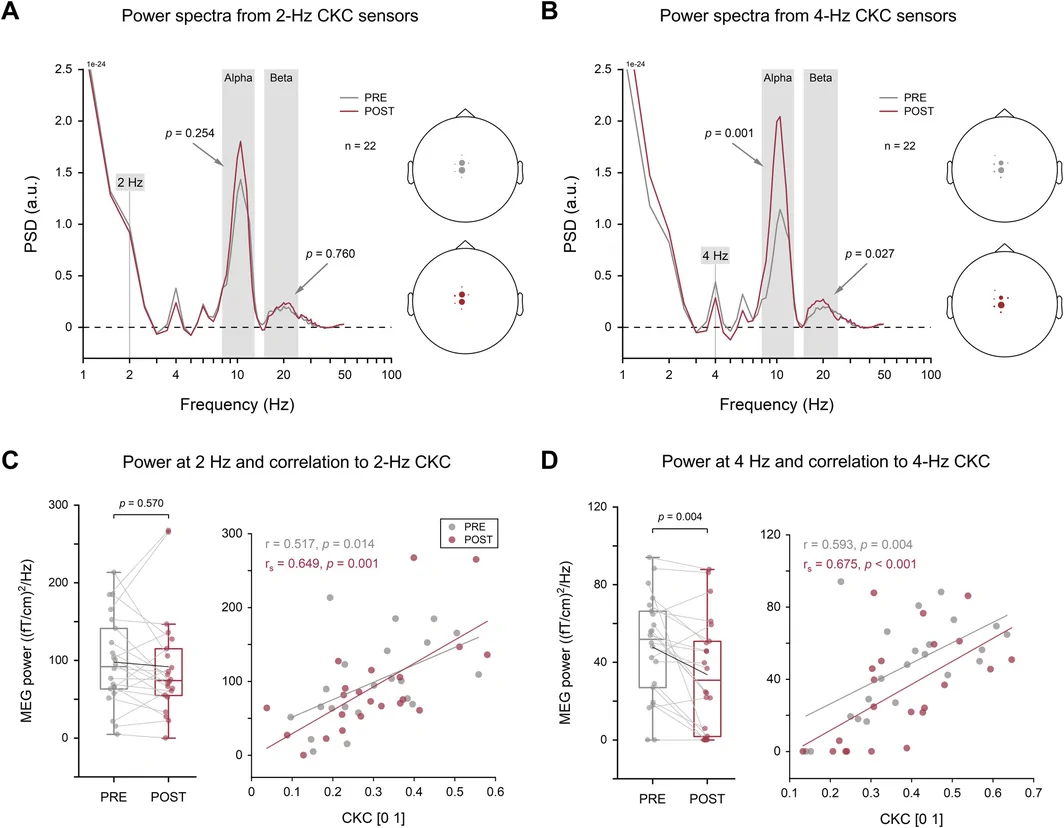
MEG power spectra extracted from the peak CKC sensors before (PRE) and after fatigue (POST). (A) Grand averages of power spectra (*n* = 22) from 2‐Hz CKC sensors. The alpha (8–13 Hz) and beta bands (15–25 Hz) are highlighted with gray bars. The location of the sensors is marked with dots, with a larger dot indicating a greater number of sensors here. (B) Grand averages of power spectra (*n* = 22) from 4‐Hz CKC sensors. (C) MEG power at 2 Hz and its correlation to 2‐Hz CKC. (D) MEG power at 4 Hz and its correlation to 4‐Hz CKC.

In addition, the power in alpha (8–13 Hz) and beta bands (15–25 Hz) was also obtained from peak CKC sensors. For 2‐Hz CKC sensors, no significant differences were observed between PRE and POST in either alpha or beta power. However, for 4‐Hz CKC sensors, both alpha and beta power were significantly stronger in POST than in PRE.

### 3.5 MEG Power Modulation to Muscle Fatigue in Peak Alpha and Beta Sensors

As shown in Figure 6, the topographic distributions of alpha‐ (8–13 Hz) and beta‐band (15–25 Hz) mean power were plotted across all gradiometer pairs, as well as their differences between PRE and POST. Somewhat surprisingly, the most prominent lower‐limb fatigue‐induced changes in MEG power were observed in the sensors above the SM1 hand regions for both alpha and beta bands. To characterize these findings in more detail, we conducted exploratory analyses by selecting four gradiometer pairs with the largest power difference above the SM1 cortex contralateral and ipsilateral to the movement, respectively, and then averaged their power spectra. Based on exploratory findings, average alpha power was significantly stronger in POST compared with PRE in both contralateral and ipsilateral hand regions. For average beta power, the ipsilateral hand region was significantly stronger in POST than in PRE; however, the contralateral was not.

**FIGURE 6:**
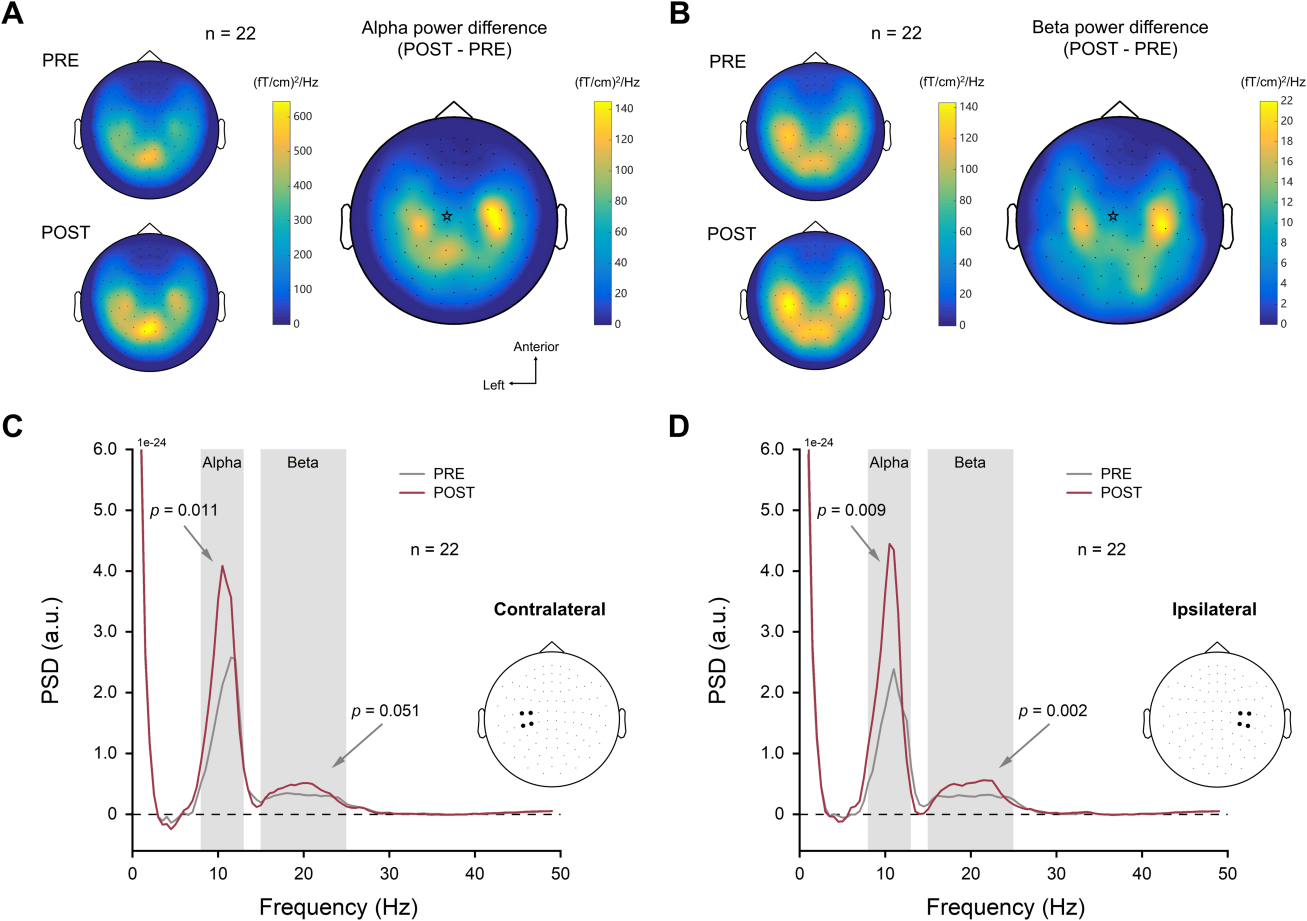
Grand average topographies of alpha (8–13 Hz) and beta (15–25 Hz) power during movement stimulation (*n* = 22) and the grand averages of power spectra extracted from the peak alpha and beta sensors before (PRE) and after fatigue (POST). (A) The topographies of alpha power and their difference between PRE and POST across all gradiometer pairs. The PRE values were always subtracted from the POST. The CKC node is shown as a star. (B) The topographies of beta power and their difference between PRE and POST across all gradiometer pairs. (C) Grand averages of average power spectra from four contralateral (left) gradiometer pairs. (D) Grand averages of average power spectra from four ipsilateral (right) gradiometer pairs.

## 4 Discussion

Our fatigue protocol induced clear muscle fatigue in GM and SOL muscles, that is, the plantar flexors. The proprioceptive stimulation (i.e., passive ankle rotations) evoked clear cortical responses to somatosensory afference, as expected. Contrary to our hypothesis, the early processing of cortical proprioceptive afference, that is, CKC strength and MEF amplitude, was not significantly affected by muscle fatigue, although the MEG power at first harmonic (4 Hz) of the movement frequency was reduced by the fatigue. However, following muscle fatigue, MEG power in the alpha and beta bands was enhanced within a multinode SM1 neuronal network.

### 4.1 Muscle Fatigue Negligibly Affects Cortical Proprioceptive Processing

As expected, the evoked passive ankle rotations led to strong CKC with underlying sources located in the contralateral SM1 somatotopic foot region (J. Chen et al. 2025; Giangrande et al. 2024; Piitulainen et al. 2015; Piitulainen, Seipäjärvi, et al. 2018). When stretching relaxed muscles, spindle Ia afferents encode the changes in fascicle length (G. Macefield and Knellwolf 2018) and muscle force (Blum et al. 2017). During sustained fatiguing isometric contraction, the majority of muscle spindles show progressive decline in their firing rate over time (G. Macefield et al. 1991). Their results suggest that fatigue may impair the peripheral proprioceptive afference, that is, muscle spindle responsiveness. In fact, this fatigue effect may depend on the muscle contraction paradigm being tested. During high‐intensity intermittent isometric contractions, superimposed tendon vibration, which provides additional burst of proprioceptive afference, is not able to alleviate muscle fatigue (Timbert et al. 2025). However, because the vibration imposes nonphysiological proprioceptive feedback, the absence of functional benefits during fatiguing exercise does not necessarily indicate impaired spindle afference. In addition, reduced stretch reflex sensitivity observed in fatigued muscles may indicate the weakening of spindle responsiveness, spindle sensitivity, and fusimotor support by γ‐motoneuron drive, all of which potentially explain the reduced spinal excitability (Avela et al. 1999). Although we did not observe changes in CKC and MEF after fatigue, the peripheral spindle responsiveness could still modify the timing and consistency of proprioceptive afference reaching the cortex. Nevertheless, it appears that the impaired spindle function partly explains the fatigue‐related impact on sensorimotor processing. The peripheral asynchrony may also lead to asynchronous activation of the cortical neurons, impairing the MEG signal power and thus CKC strength. Therefore, there is indeed cortical adaptation that compensates for altered peripheral somatosensory feedback to facilitate intact sensorimotor integration. Moreover, the intensity of proprioceptive stimuli, for example, larger versus smaller movement range (Nurmi et al. 2023), did not significantly affect CKC strength, provided that the regularity of stimulation sequence is maintained (Mujunen et al. 2021). This highlights the “invariance” of early proprioceptive processing, indicating that the amount of proprioceptive afference reaching the cortex is finite—only requiring attainment of the threshold for triggering cortical responses (Mima et al. 1996; Seiss et al. 2002).

CKC is theoretically interpreted as a frequency‐dependent measure of phase coupling between limb kinematics and cortical activity (Bourguignon et al. 2019). In fact, MEG signals mainly consist of inherent sustained electrophysiological “brain noise” and some external noise. A simulation experiment has shown that improved signal‐to‐noise ratio may enhance the coherence strength in noisy signals (Muthukumaraswamy and Singh 2011). Because CKC strength typically has positive correlation with sustained MEF amplitude (J. Chen et al. 2025; Mujunen et al. 2021; Piitulainen, Illman, et al. 2018), amplitude coupling also plays an important role in the coherence estimation, especially in noisy signals like MEG. As expected, we observed significant positive correlation between CKC strength and MEF amplitude, but it was significant only before muscle fatigue, not after. Although MEF amplitude was not significantly altered following muscle fatigue, a small effect between PRE and POST suggested that MEF changes (to be lower) did exist. In addition, 2‐ and 4‐Hz MEG power were consistently positively correlated with CKC strength both before and after fatigue. It is noteworthy that, as 4‐Hz MEG power was reduced, the CKC would have been expected to decrease as well, based on the relationship between MEG signal‐to‐noise ratio and coherence values. Conversely, the fact that CKC did not decrease may indicate that it “relatively” increased physiologically, yet the rise in brain noise compromised the coherence estimate.

Regarding subcortical factors, muscle fatigue can alter the cerebello‐thalamo‐cortical network, specifically by weakening the cerebellar inhibition onto the SM1 cortex (Casamento‐Moran et al. 2023). As reduced cerebellar inhibition correlates with less perceived fatigue, this fatigue‐induced disinhibition appears to reflect a compensatory response. Nevertheless, cortical proprioceptive processing remains functionally insulated from such subcortical fluctuations, consistent with recent evidence that acute exogenous modulation of cerebellar inhibition does not alter the SM1 excitability and its proprioceptive processing (i.e., CKC) in either healthy individuals or cerebellar ataxia (Georgiev et al. 2026). We further expect the cerebello‐thalamo‐cortical pathway to play a key regulatory role in acute fatigue during sustained voluntary contraction, as predictive feedforward control of the cerebellum dynamically evaluates somatosensory input and motor output to adapt to fatigued states (Liu et al. 2003; Shadmehr et al. 2010).

### 4.2 SM1 Cortical Power Is Regionally Modulated by Muscle Fatigue

For rhythmic 2‐Hz passive movement, each cycle of ankle rotation generates two primary afferent proprioceptive bursts—one at the onset of the dorsiflexion phase and one at the onset of the plantarflexion phase—due to the stretching of the plantarflexor and dorsiflexor muscles, respectively, leading to MEG power accumulation at 4 Hz. We found that MEG power at 4 Hz was reduced by ~30% following muscle fatigue, which implies smaller and/or less synchronized cortical neuronal population involved in the cortical proprioceptive processing—potentially due to more asynchronous somatosensory feedback generation, conduction, and processing. At the same sensor location, increased alpha and beta power indicates stronger neuronal synchronization in the alpha and beta bands (Jensen et al. 2005; Jensen and Mazaheri 2010), possibly shifting away from harmonic frequencies of the movement. Because the CKC was preserved after fatigue, it is most likely that the fatigue‐induced asynchrony in the peripheral somatosensory feedback to the cortex is compensated by recruiting more cortical neurons for somatosensory processing.

Lower‐limb proprioceptive stimulation has been shown to activate the SM1 cortex beyond the expected foot region (F. Li et al. 2027; Mujunen et al. 2025). Our SM1 power results revealed the most prominent fatigue‐related increase of alpha and beta power was lateral to the foot somatotopy, peaking in the sensors above bilateral hand regions. These findings suggest that fatigue‐induced power modulations are not confined to a particular effector region but affects SM1 cortex dynamics on a wider basis. One explanation might be that the Rolandic foot area exhibits inherently weaker MEG sensitivity compared with the hand (Hillebrand and Barnes 2002), as its cortical representation within the medial wall of the paracentral lobule is anatomically smaller and situated both deeper and more medially (Ciccarelli et al. 2005; Dobkin et al. 2004; Francis et al. 2009). Importantly, it has recently been suggested that, in addition to effector‐specific regions for isolating fine action, intereffector regions exist to integrate information from body movements (Gordon et al. 2023). In the present study, the alpha and beta power changes were observed at the sensor level with bilateral peaks above the intereffector regions. This result supported that the inhibitory control of sensorimotor neuronal populations, that is, their synchronization activity in alpha or beta band, is enhanced in the fatigued state, which is reflected by increased alpha or beta power (Jensen et al. 2005; Jensen and Mazaheri 2010). This region‐specific power modulation relates to inhibitory neurotransmitter GABA release, as evidenced by the increased GABA concentration in hand regions of the sensorimotor cortex after lower‐limb exercise (Coxon et al. 2018).

Furthermore, we propose that muscle fatigue strengthens localized “inhibitory stencils” to constrain cortical processing in noneffector regions (Z. Chen et al. 2026). This allows the processing of proprioceptive information to be more concentrated in the effector region, that is, CKC node, in which alpha and beta activity is relatively weak and stable. The beta power in our results showed bilateral increases in SM1 cortex after fatigue, which is consistent with the resting‐state results from a grip fatigue study (Matta et al. 2025). As proposed by the “status quo” hypothesis, beta activity reflects the maintenance of the current sensorimotor state, which means that higher beta power results in greater difficulty in releasing cortical inhibition to initiate movement (Barone and Rossiter 2021; Engel and Fries 2010). Specifically, the motor cortex is less active once the peripheral proprioceptors become exhausted or when the SM1 cortex itself is depleted as well. Indeed, upregulation of beta power has been linked to impaired motor function, such as less motor flexibility (Pierrieau et al. 2025). Moreover, impaired movement execution observed in aging (Rossiter et al. 2014) and Parkinson's disease (Little and Brown 2014) is directly related to higher beta power at rest. Similarly, diabetic sensorimotor peripheral neuropathy leads to higher resting beta power, along with more widespread cortical activation when proprioceptive afference reaches the SM1 (Mujunen et al. 2025). Taken together, the power modulation of cortical responses to muscle fatigue may be regionally specific as opposed to a global phenomenon.

### 4.3 Limitations and Future Perspectives

In comparison with proprioceptive stimulation with a long interstimulus interval (ISI ~ 3 s; Smeds et al. 2017), acute exercise‐induced fatigue is more capable of rapidly collecting sufficient stimuli for higher signal‐to‐noise ratio. It is noteworthy that rapid rhythmic stimulation may mask some fatigue‐related effects on proprioceptive signals, because the temporal expectation of regularities can boost the neural processing of anticipated movements (Nobre and van Ede 2018; Rohenkohl et al. 2012), and even result in time‐locked cortical responses to omitted stimuli (Andersen and Lundqvist 2019) given that the interstimulus interval is stable.

Movement‐related artifacts derived from rhythmic ankle‐joint rotations were sometimes observed at the lowermost MEG helmet sensors (in 6/24 participants) and were similarly reported in prior lower‐limb CKC studies (J. Chen et al. 2025). Nevertheless, the presence of distinct CKC peaks at the vertex sensors located above the SM1 cortex suggests that the artifacts did not mask the actual neurophysiological responses. It appears that continuous lower‐limb movement stimuli may produce unintended “oscillating” head movements when compared with the intermittent ones (Mujunen et al. 2022). If necessary, individualized MRI‐based head casts could be used to reduce fluctuations of the head position (Meyer et al. 2017). We also recommended to employ a moderate movement range and angular velocity for ankle rotation, in conjunction with precise alignment of the ankle joint and actuator rotation axes, which can effectively avoid any redundant movements.

Due to the reset of proprioceptive stimulator, we could not avoid few minutes recovery period between the fatigue tasks and subsequent proprioceptive stimulation. It has been reported that, despite rapid recovery of MVC force within few minutes, resting twitch forces evoked by motor nerve stimulation do not recover appreciably within 20 min after sustained low‐intensity contractions (Carroll et al. 2017; Søgaard et al. 2006). We recommend that caution should remain when estimating the recovery time and extent of fatigue, given the task‐dependent nature of fatigue (Enoka et al. 2011) and its dependence on the measures (Krüger et al. 2019). Future studies should pay attention to the fatigue recovery or directly track cortical proprioceptive processing as fatigue evolves.

## 5 Conclusion

Cortical proprioceptive responses to passive ankle‐joint movements were negligibly affected by muscle fatigue. Intriguingly, fatigue‐induced increases in alpha and beta power were observed, extending beyond the SM1 foot region to the bilateral hand regions. This observation can be interpreted as region‐specific cortical inhibition, which may facilitate more focused lower‐limb proprioceptive processing, thus compensating for the asynchrony of spindle proprioceptive afference caused by fatigue. It is recommended that future studies investigate the patterns of cortical proprioceptive processing as fatigue evolves, and their implications for patients with chronic pathological fatigue (Chaudhuri and Behan 2004).

## Author Contributions

**Junru Chen:** conceptualization, methodology, software, data curation, investigation, validation, formal analysis, visualization, project administration, writing – original draft. **Toni Mujunen:** methodology, software, data curation, writing – review and editing. **Feiyue Li:** investigation, writing – review and editing. **Riku Nikander:** writing – review and editing, supervision. **Harri Piitulainen:** conceptualization, methodology, supervision, resources, funding acquisition, writing – review and editing, project administration.

## Conflicts of Interest

The authors declare no conflicts of interest.

## Data Availability

The data that support the findings of this study are available from the corresponding author upon reasonable request.
